# Supplementary material for: Anti-Quorum Sensing Activities of Gliptins against Pseudomonas aeruginosa and Staphylococcus aureus
Source: Biomedicines. 2022 May 18;10(5):1169. doi: 10.3390/biomedicines10051169 (PMC9138634; doi:10.3390/biomedicines10051169)
Supplement: Supplementary file 1 [file biomedicines-10-01169-s001.zip › biomedicines-1720698-supplementary.pdf]

"Supplementary Data"

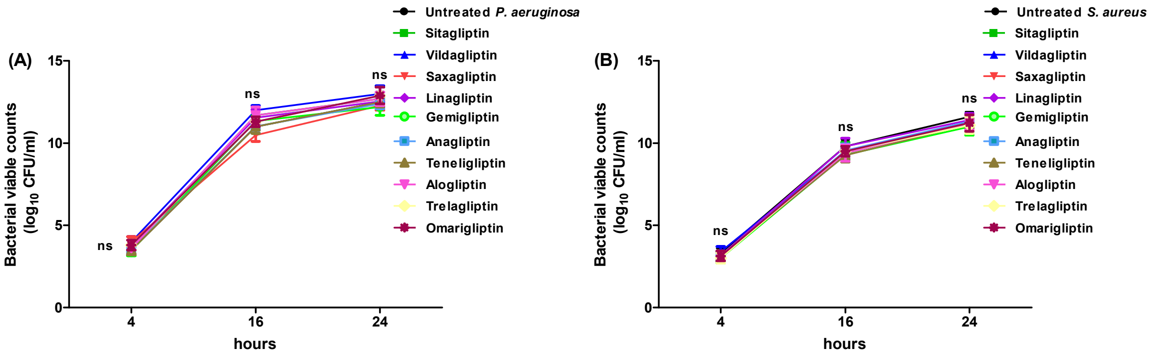

Figure S1. Viable count of bacterial cells in the presence and absence of the tested gliptins at different time periods. There was no significant difference between bacterial counts in the presence or absence of tested gliptins.

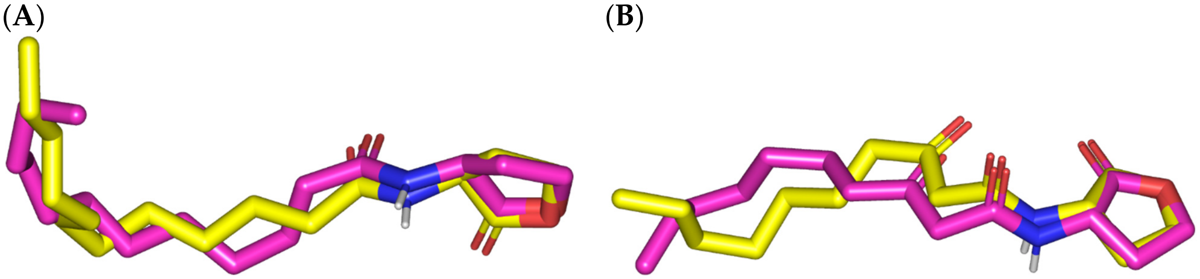

Figure S2. Superimposing the co-crystallized (magenta sticks) and redocked (yellow sticks) ligands at *Pseudomonas aeruginosa* quorum-sensing transcription proteins QscR (**A**) and LasR (**B**) for validating the adopted directed docking protocol.

Table S1. Descriptive *Pseudomonas aeruginosa* LasI-ligand binding interactions via docking protocol.

**Compound**	**Ligand-target interaction description**<br>**[Type; Length (Å); Angle (°); Binding Residues]**
**SIT**	H-bond **;** 2.6 **Å ;** 127 ° **;** Arg30<br>H-bond **;** 3.1 **Å ;** 125 ° **;** Arg104<br>H-bond **;** 2.3 **Å ;** 139 ° **;** Arg104<br>π-π interaction ; 2.7 **Å ;** Trp33<br>π-H interaction ; 3.4 **Å ;** Phe27<br>π-H interaction ; 2.5 **Å ;** Trp69
**OMR**	H-bond **;** 3.1 **Å ;** 129 ° **;** Arg30<br>H-bond **;** 3.3 **Å ;** 135 ° **;** Arg104<br>π-π interaction ; 2.1 **Å ;** Trp33

	π-H interaction ; 3.5 **Å ;** Phe27<br>π-H interaction ; 1.4 **Å ;** Trp69
**LIN**	H-bond **;** 2.4 **Å ;** 130 ° **;** Arg30<br>H-bond **;** 1.9 **Å ;** 147 ° **;** Arg30<br>H-bond **;** 2.2 **Å ;** 121 ° **;** Thr142<br>H-bond **;** 3.4 **Å ;** 141 ° **;** Thr144<br>π-H interaction ; 2.3 **Å ;** Trp33<br>π-H interaction ; 1.9 **Å ;** Trp69
**TRG**	H-bond **;** 2.4 **Å ;** 133 ° **;** Arg30<br>H-bond **;** 3.3 **Å ;** 158 ° **;** Phe105<br>π-H interaction ; 3.4 **Å ;** Phe27<br>π-H interaction ; 2.1 **Å ;** Trp33
**TZD-C8**	H-bond **;** 1.8 **Å ;** 123 ° **;** Arg30<br>H-bond **;** 3.4 **Å ;** 132 ° **;** Ile170

Table S2. Descriptive *Pseudomonas aeruginosa* QscR-ligand binding interactions via docking protocol.

**Compound**	**Ligand-target interaction description [Type; Length (Å); Angle (°); Binding Residues]**
**SIT**	H-bond ; 1.7 **Å** ; 129 ° ; Ser38<br>H-bond ; 2.6 **Å** ; 154 ° ; Tyr66<br>H-bond ; 2.3 **Å** ; 124 ° ; Asp75<br>π-π interaction ; 5.1 **Å** ; Phe39<br>π-π interaction ; 3.4 **Å** ; Trp90<br>π-H interaction ; 3.2 **Å** ; Tyr66<br>van der Waals ; 4.3 **Å** ; Arg42 sidechain C*β*
**OMR**	Polar ; 1.7 **Å** ; Asp75<br>H-bond ; 3.3 **Å** ; 124 ° ; Ser38<br>H-bond ; 3.4 **Å** ; 129 ° ; Tyr58<br>H-bond ; 2.1 **Å** ; 136 ° ; Trp62<br>H-bond ; 2.4 **Å** ; 139 ° ; Met127<br>π-H interaction ; 3.5 **Å** ; Phe39<br>π-H interaction ; 3.3 **Å** ; Tyr52<br>π-π interaction ; 4.5 **Å** ; Tyr66<br>van der Waals ; 4.2 **Å** ; Arg42 sidechain C*β*
**LIN**	Polar ; 2.6 **Å** ; Tyr66<br>Polar ; 2.8 **Å** ; Asp75<br>H-bond ; 2.8 **Å** ; 120 ° ; Ser38<br>H-bond ; 2.7 **Å** ; 127 ° ; Gly40<br>H-bond ; 3.0 **Å** ; 139 ° ; Ser129<br>π-π interaction ; 4.3 **Å** ; Phe54<br>π-H interaction ; 3.4 **Å** ; Tyr58<br>π-H interaction ; 3.5 **Å** ; Tyr66<br>π-H interaction ; 3.6 **Å** ; Trp102<br>van der Waals ; 4.6 **Å** ; Arg42 sidechain C*β*
**TRG**	Polar ; 3.1 **Å** ; Ser38<br>H-bond ; 3.3 **Å** ; 126 ° ; Trp62<br>H-bond ; 3.3 **Å** ; 129 ° ; Tyr66<br>π-π interaction ; 3.6 **Å** ; Phe54<br>π-π interaction ; 3.6 **Å** ; Tyr66
**Q9**	H-bond ; 2.8 **Å** ; 146 ° ; Ser38<br>H-bond ; 3.2 **Å** ; 128 ° ; Trp58<br>H-bond ; 2.0 **Å** ; 161 ° ; Asp75<br>π-π interaction ; 3.9 **Å** ; Phe54<br>π-H interaction ; 3.5 **Å** ; Tyr66

Table S3. Descriptive *Pseudomonas aeruginosa* LasR-ligand binding interactions via docking protocol.

Compound	Ligand-target interaction description<br>[Type; Length (Å); Angle (°); Binding Residues]
**SIT**	H-bond ; 1.7 **Å** ; 155 ° ; Trp60<br>H-bond ; 2.5 **Å** ; 124 ° ; Trp60<br>H-bond ; 3.4 **Å** ; 128 ° ; Arg61<br>H-bond ; 3.0 **Å** ; 146 ° ; Asp73<br>H-bond ; 2.0 **Å** ; 144 ° ; Tyr93<br>H-bond ; 3.1 **Å** ; 160 ° ; Ser129<br>π-H interaction ; 3.9 **Å** ; Tyr47<br>π-H interaction ; 4.0 **Å** ; Phe101
**OMR**	Polar ; 3.0 **Å** ; Asp73<br>H-bond ; 2.2 **Å** ; 132 ° ; Tyr56<br>H-bond ; 3.4 **Å** ; 128 ° ; Arg61<br>H-bond ; 2.5 **Å** ; 127 ° ; Ser129<br>π-π interaction ; 3.5 **Å** ; Tyr47<br>π-H interaction ; 5.0 **Å** ; Phe101
**LIN**	Polar ; 3.4 **Å** ; Ser129<br>H-bond ; 3.1 **Å** ; 146 ° ; Tyr56<br>H-bond ; 2.3 **Å** ; 147 ° ; Trp60<br>H-bond ; 2.4 **Å** ; 146 ° ; Asp73<br>π-H interaction ; 4.0 **Å** ; Tyr47<br>π-H interaction ; 3.4 **Å** ; Tyr56
**TRG**	H-bond ; 3.4 **Å** ; 156 ° ; Tyr56<br>H-bond ; 3.3 **Å** ; 126 ° ; Arg61
**Q9**	H-bond ; 1.9 **Å** ; 158 ° ; Tyr56<br>H-bond ; 1.7 **Å** ; 168 ° ; Asp73<br>H-bond ; 2.7 **Å** ; 126 ° ; Tyr93<br>H-bond ; 1.7 **Å** ; 163 ° ; Ser129<br>π-H interaction ; 4.2 **Å** ; Tyr47

Table S4. Descriptive *Staphylococcus aureus* AgrC ligand-binding interactions via docking protocol.

Compound	Ligand-target interaction description<br>[Type; Length (Å); Angle (°); Binding Residues]
**SIT**	H-bond ; 2.1 **Å** ; 121 ° ; Asp374<br>H-bond ; 3.2 **Å** ; 126 ° ; Asp374<br>H-bond ; 3.3 **Å** ; 126 ° ; Asp374<br>H-bond ; 1.6 **Å** ; 162 ° ; Ser387<br>H-bond ; 3.0 **Å** ; 125 ° ; Gln423<br>π-H interaction ; 3.9 **Å** ; Ile415<br>van der Waals ; 4.1 **Å** ; Asn339 sidechain C*β*
**LIN**	Polar ; 3.2 **Å** ; Asp374<br>H-bond ; 3.4 **Å** ; 128 ° ; Ile375<br>H-bond ; 2.8 **Å** ; 124 ° ; Arg377<br>H-bond ; 3.3 **Å** ; 123 ° ; Arg377<br>H-bond ; 3.4 **Å** ; 147 ° ; Leu381<br>π-H interaction ; 4.3 **Å** ; Ile415<br>π-H interaction ; 4.7 **Å** ; Leu381<br>van der Waals ; 4.3 and 4.5 **Å** ; Asn339 sidechain C*β* and C*δ*<br>van der Waals ; 4.6 **Å** ; Gln423 sidechain C*β*
**TRG**	Polar ; 3.3 **Å** ; Asp374<br>Polar ; 3.4 **Å** ; Asp374<br>H-bond ; 3.1 **Å** ; 124 ° ; Asp374<br>H-bond ; 2.2 **Å** ; 128 ° ; Arg377<br>H-bond ; 3.3 **Å** ; 129 ° ; Arg377<br>H-bond ; 3.4 **Å** ; 129 ° ; Ser387<br>π-H interaction ; 4.1 **Å** ; Leu381
**OMR**	Polar ; 3.4 **Å** ; Glu391<br>H-bond ; 2.1 **Å** ; 128 ° ; Asp373<br>H-bond ; 3.3 **Å** ; 127 ° ; Asp374<br>π-H interaction ; 3.9 **Å** ; Ile415<br>π-H interaction ; 3.7 **Å** ; Ile375
**ADP-*β*-N**	Polar ; 3.3 **Å** ; Ser387<br>Polar ; 3.4 **Å** ; Gln423<br>H-bond ; 3.2 **Å** ; 128 ° ; Asp373<br>H-bond ; 2.5 **Å** ; 135 ° ; Gly392<br>H-bond ; 1.8 **Å** ; 129 ° ; Gly392<br>H-bond ; 3.4 **Å** ; 128 ° ; Arg393<br>H-bond ; 2.0 **Å** ; 140 ° ; Leu397<br>H-bond ; 2.9 **Å** ; 125 ° ; Gln423<br>π-π interaction ; 5.2 **Å** ; Phe382<br>π-H interaction ; 3.1 **Å** ; Leu397

Table S5. Descriptive *Staphylococcus aureus* AgrA ligand-binding interactions via docking protocol.

<table>
<tr><th rowspan="2">Compound</th><th colspan="2">Ligand-target interaction description
[Type; Length (Å); Angle (°); Binding Residues]</th></tr>
<tr><th></th><th></th></tr>
<tr><td>SAV</td><td><u>Site I</u>
H-bond ; 2.5 Å ; 133 ° ; Arg218
H-bond ; 2.5 Å ; 132 ° ; Arg233
H-bond ; 3.3 Å ; 127 ° ; Arg233
π-H interaction ; 2.8 Å ; His200
π-H interaction ; 3.6 Å ; His200
π-H interaction ; 4.8 Å ; Asn201
π-H interaction ; 3.9 Å ; Tyr229</td><td><u>Site II</u>
H-bond ; 3.4 Å ; 163 ° ; Lys187
π-H interaction ; 4.8 Å ; His169</td></tr>
<tr><td>SIT</td><td><u>Site I</u>
H-bond ; 2.0 Å ; 155 ° ; Arg218
H-bond ; 2.6 Å ; 144 ° ; Val232
H-bond ; 2.4 Å ; 129 ° ; Arg233
H-bond ; 3.3 Å ; 143 ° ; Asn201
π-H interaction ; 5.0 Å ; His200</td><td><u>Site II</u>
H-bond ; 2.7 Å ; 146 ° ; His169
H-bond ; 2.6 Å ; 142 ° ; Leu186
H-bond ; 2.9 Å ; 148 ° ; Arg198
π-H interaction ; 2.7 Å ; Ser164</td></tr>
<tr><td>LIN</td><td><u>Site I</u>
H-bond ; 3.2 Å ; 128 ° ; Arg233
H-bond ; 2.8 Å ; 129 ° ; Arg233
H-bond ; 2.7 Å ; 128 ° ; His200
π-π interaction ; 4.9 Å ; Tyr229</td><td><u>Site II</u>
Polar ; 2.9 Å ; Asn201
H-bond ; 3.4 Å ; 168 ° ; His168
π-π interaction ; 4.1 Å ; His168</td></tr>
<tr><td>TRG</td><td><u>Site I</u>
Polar ; 3.3 Å ; Asn201
H-bond ; 2.2 Å ; 140 ° ; Arg233
H-bond ; 3.3 Å ; 156 ° ; Arg233
H-bond ; 2.4 Å ; 124 ° ; His200
π-π interaction ; 4.8 Å ; His200</td><td><u>Site II</u>
H-bond ; 3.3 Å ; 159 ° ; His169
H-bond ; 3.3 Å ; 146 ° ; Gly184
H-bond ; 2.6 Å ; 165 ° ; Leu186
π-π interaction ; 4.3 Å ; His169</td></tr>
<tr><td>OMR</td><td><u>Site I</u>
H-bond ; 2.2 Å ; 140 ° ; Arg218
H-bond ; 2.7 Å ; 134 ° ; Arg218
H-bond ; 3.4 Å ; 121 ° ; Arg233</td><td><u>Site II</u>
H-bond ; 2.4 Å ; 157 ° ; His169
π-π interaction ; 5.0 Å ; His169</td></tr>
</table>
